# Mortality and years of life lost related to adverse drug events in Brazil

**DOI:** 10.11606/s1518-8787.2024058005458

**Published:** 2024-04-25

**Authors:** Lunara Teles Silva, Ana Carolina Figueiredo Modesto, Rodrigo Alves de Oliveira, Rita Goreti Amaral, Flavio Marques Lopes

**Affiliations:** I Universidade Federal de Goiás Faculdade de Medicina Programa de Pós-Graduação em Ciências da Saúde Goiânia GO Brasil Universidade Federal de Goiás. Faculdade de Medicina. Programa de Pós-Graduação em Ciências da Saúde. Goiânia, GO, Brasil; II Universidade Federal de Goiás Hospital das Clínicas Goiânia GO Brasil Universidade Federal de Goiás. Hospital das Clínicas. Goiânia, GO, Brasil; III Tribunal Regional do Trabalho da 18ª Região Goiânia GO Brasil Tribunal Regional do Trabalho da 18ª Região. Goiânia, GO, Brasil; IV Universidade Federal de Goiás Faculdade de Farmácia Goiânia GO Brasil Universidade Federal de Goiás. Faculdade de Farmácia. Goiânia, GO, Brasil

**Keywords:** Drug-Related Side Effects and Adverse Reactions, Pharmacoepidemiology, Databases, Pharmaceutical

## Abstract

**OBJECTIVE:**

To assess regional and national mortality and years of life lost (YLL) related to adverse drug events in Brazil.

**METHODS:**

This is an ecological study in which death records from 2009 to 2018 from the Mortality Information System were analyzed. Codes from the International Classification of Diseases 10^th^ revision (ICD-10) that indicated drugs as the cause of death were identified. The number of deaths and the YLL due to adverse drug events were obtained. Crude, age- and gender-specific, and age-adjusted mortality rates and YLL rates per 100,000 inhabitants were formed by year, age group, gender, and Brazilian Federative Unit. Rate ratios were calculated by comparing rates from 2009 to 2018. A joinpoint regression model was applied for temporal analysis.

**RESULTS:**

For the selected ICD-10 codes, a total of 95,231 deaths and 2,843,413 YLL were recorded. Mortality rates from adverse drug events increased by a mean of 2.5% per year, and YLL rates increased by 3.7%. Increases in rates were observed in almost all age groups for both genders. Variations in rates were found between Federative Units, with the highest age-adjusted mortality and YLL rates occurring in the Distrito Federal.

**CONCLUSIONS:**

The numbers and rates of deaths and YLL increased during the study period, and variations in rates of deaths and YLL were observed between Brazilian Federative Units. Information on multiple causes of death from death certificates can be useful for quantifying adverse drug events and analyzing them geographically, by age and by gender.

## INTRODUCTION

Drug therapy has been recognized as a determinant of the population’s health in two different ways. First, the benefits of pharmacotherapy in recovering and improving health quality have established drugs as one of the main therapeutic approaches^1,2^. Second, the benefits of drug use are often accompanied by potential harm. The issues caused by drugs, i.e. adverse drug events (ADEs), have been recognized as a significant public health problem and a major cause of death^2,3^. In the United States (US), the annual rates of deaths due to adverse drug reactions (ADRs), a subtype of ADEs, ranged from 0.08 to 0.12 per 100,000 inhabitants over an eight-year study period^4^. Regarding accidental drug poisoning, in the US, there has been an increase of more than 6% in the mean number of deaths each year, from 2,475 deaths in 1979 to 38,675 deaths in 2014^5^. Virtually all drugs can cause or contribute to a fatal event^6^. For example, psychotropic drugs and antidepressants have been linked to the risk of sudden cardiac death^7^, and corticosteroids, not usually associated with fatal events, can contribute to the development of potentially fatal diseases such as diabetes mellitus^6^.

Cause of death is one of the simplest approaches to measuring health problems in a population and obtaining traditional epidemiological measures such as mortality rates and years of life lost (YLL), both of which are fundamental for establishing public health priorities^10,11^. YLL is a valuable indicator for quantifying premature mortality and monitoring trends over the years^12^. One way to access national estimates of deaths is to use vital statistics records, such as death certificates, which can identify the cause(s) of death, coded by the International Classification of Diseases (ICD). To our knowledge, there is only one outdated study in Brazil that applied a methodology using vital records to identify potential YLL related to deaths due to all intentional types of drug intoxication^13^. Given this knowledge gap, the aim of this study is to assess regional and national mortality and YLL related to ADEs in Brazil over a ten-year period.

## METHODS

The Strengthening the Reporting of Observational Studies in Epidemiology (STROBE) guidelines were followed for the presentation of this study.

### Study Design and Data Sources

An ecological study was conducted to estimate deaths related to ADEs in Brazil over a 10-year period (January 1, 2009, to December 31, 2018). The data source used for this study was the *Sistema de Informação sobre Mortalidade* (SIM – Mortality Information System), whose underlying data source are death certificates^14^. The dataset was obtained from the Brazilian Unified Health System Department of Informatics (DATASUS), which is managed by the Ministry of Health. DATASUS compiles data nationally and publishes it as blinded data. The registry covers deaths of individuals residing in Brazil since 1975. These reports include demographic characteristics, geographic information, and the cause of death, which is assigned using ICD codes, 10^th^ revision. Coding is conducted at the central level by trained personnel based on information reported in the death certificates, which are filled out by health workers. The demographic data for the period were collected from the Brazilian Institute of Geography and Statistics (IBGE), including population projections and life tables by year, Brazilian Federative Unit, gender, and age^15^.

### Definition of Cases

A death was classified as a case when the underlying cause of death and/or the immediate cause and/or the contributing conditions were associated with at least one ICD-10 code related to an ADE during the study period. The underlying cause of death is defined as “the disease or injury that initiated the train of morbid events leading directly to death, or the circumstances of the accident or violence that caused the fatal injury.” The immediate cause is the final disease, injury, or complication (resulting from the underlying cause) that directly causes death, and the contributing causes of death are diseases or injuries that contribute to the fatal outcome but are not related to the disease or condition directly causing death^16^.

A total of 618 codes adapted from the study by Mota et al.^17^ were used to select cases of deaths from drug poisoning, mental disorders due to drug use, and drug-induced diseases. Codes that included ADR (harm produced by the use of a drug in a recommended manner)^3^ and drug poisoning coded as unintentional or of undetermined cause were used (see supporting information)^a^.

Deaths with codes indicating intentional poisoning as the external cause, even when accompanied by other codes, were not included. After selecting the deaths based on the codes, cases in which death was coded as homicide or suicide were excluded.

### Outcome Variables

The number of deaths and the YLL due to deaths related to ADEs were considered outcomes. The YLL were calculated using life expectancy obtained from Brazilian life tables^15^, matching the individuals’ year of death, gender, and age group, and pairing the Brazilian Federative Unit where their death occurred with the reference life table, as follows:


YLL(c,g,a,y,f)=D(c,g,a,y,f)×SLE(g,a,y,f),

In which: D_(c, g, a, y, f)_ is the number of deaths due to cause c (ADE), for gender g, age a, year y, and Federative Unit f; SLE_(g, a, y, f)_ is the standard life expectancy at gender g, age a, year y, and Federative Unit f, according to Brazilian life tables^12^.

### Explanatory Variables

The variables analyzed were year of death (2009 to 2018), age at death (categorized as: ≤ 4; 5–14; 15–24; 25–34; 35–44; 45–54; 55–64; 65–74; ≥ 75), gender (female or male), and Brazilian Federative Unit where death occurred (27 units). In addition, ethnicity, marital status, education, place of death, and medical assistance were used to characterize the population.

### Statistical Analysis

Data were analyzed using R version 4.0.2 (The R Foundation for Statistical Computing, Vienna, Austria, 2020). First, a descriptive statistical analysis was performed, including frequencies, means, standard deviations (SD), medians, and interquartile ranges (IQR). The average YLL per death was estimated by dividing the total YLL by the total number of deaths. Second, the annual crude rates of deaths and YLL per 100,000 inhabitants were estimated by using the number of deaths and YLL as the numerator and the population estimates for each year as the denominator. Additionally, age-standardized rates with 95% confidence intervals (95%CI) were obtained using the direct method and adjusted for the World Standard population of 2000^18^.

Third, gender- and age-specific mortality rates and YLL per 100,000 inhabitants were estimated, along with the corresponding 95%CI (sum of deaths and YLL divided by the number of cases in the respective subgroup). Fourth, deaths were stratified geographically by the person’s place of death, one of Brazil’s 27 Federative Units. Age-standardized rates were obtained for each Brazilian Federative Unit. Time trends in overall rates by age and by gender were evaluated using the Joinpoint Regression Program Version 4.8.0.1 (Statistical Research and Applications Branch, National Cancer Institute). This tool identifies trend changes, and the joinpoint model is described using the annual percentage change (APC) during a particular trend and the average annual percentage change (AAPC) for the entire period. In addition to assessing temporal changes in mortality rates and YLL, the specific rates of the Brazilian Federative Units were also examined. The model was applied with a significance level of 5%^19^.

### Ethical Aspects

This study was based on a national database of causes of death, which contains anonymous information and is available to the public. Nevertheless, ethical approval was obtained from the Research Ethics Committee of the Universidade Federal de Goiás (CAEE 32355720.9.0000.5083).

## RESULTS

### Mortality

A total of 95,231 deaths related to ADEs in Brazil were recorded in the mortality database over a 10-year analysis, corresponding to 0.78% of all deaths. When specifically considering the underlying cause of death, 32,672 cases related to ADEs were found (0.27% of all deaths in Brazil and 34.31% of deaths with at least one code appearing on death certificates). The mean number of deaths per year was 9,523.1 (SD 1,427.94), ranging from a minimum of 7,060 in 2009 to a maximum of 11,481 in 2018 (an average annual increase of 5.5%). For both females and males, the majority of deaths occurred in the ≥ 75 years age group, with 10,267 and 9,398 cases, respectively. The median age of death was 57.00 (IQR = 37.00–72.00). Table 1 details the social characteristics and conditions of death in the study population.

**Table 1:** Descriptive analysis of individuals who died due to cases related to adverse drug events in Brazil and conditions of death. Brazil, 2009–2018 (n = 95,231).

Characteristics	Frequency n (%)
Gender	
Woman	41,013 (43.07)
Man	54,197 (56.91)
Other or missing data	21 (0.02)
Age group (years)	
0–4	3,458 (3.63)
5–14	2,179 (2.29)
15–24	6,421 (6.74)
25–34	8,685 (9.12)
35–44	10,581 (11.11)
45–54	12,876 (13.52)
55–64	15,499 (16.28)
65–74	15,760 (16.55)
≥ 75	19,671 (20.66)
Other or missing data	101 (0.11)
Ethnicity	
White	50,106 (52.62)
Black	7,678 (8.06)
Yellow	641 (0.67)
Mixed-race	32,424 (34.05)
Indigenous	180 (0.19)
Other or missing data	4,202 (4.41)
Marital status	
Single	31,738 (33.33)
Married	32,105 (33.71)
Widowed	13,347 (14.02)
Divorced	6,075 (6.38)
Consensual union	2,243 (2.36)
Other or missing data	9,723 (10.21)
Education (number of years)	
No education	8,730 (9.18)
1–3	19,431 (20.40)
4–7	20,432 (21.46)
8–11	16,647 (17.48)
≥ 12	7,629 (8.01)
Other or missing data	22,362 (23.48)
Place of death	
Hospital	81,191 (85.26)
Other health service	3,911 (4.11)
Household	7,471 (7.85)
Public via	1,146 (1.20)
Other or missing data	1,512 (1.59)
Health assistance	
Yes	51,581 (54.16)
No	3,902 (4.10)
Other or missing data	39,748 (41.74)

The average annual crude mortality rate due to ADEs per 100,000 inhabitants was 4.73 (95%CI: 4.30–5.15) for overall deaths. During the study years, the highest age-adjusted overall mortality rate was observed in 2018 (5.06 per 100,000 population, 95%CI: 4.97–5.15), and there was an annual increase of 2.5% in the rate over the years. The joinpoint analysis for the total population identified a change point in 2013, resulting in two segments in the analysis: the first one (2009–2013) with the fastest growth in the mortality trend and the second one with a slower increase. According to the age-adjusted rates, the period showed an annual increase in mortality. Table 2 shows the complete data on the rates over the years.

**Table 2:** Number of deaths, years of life lost, crude mortality rates by adverse drug event per 100,000 inhabitants, and age-adjusted mortality rates by ADE. Brazil, 2009–2018.

Year	Population^a^	Number of deaths	Crude mortality rate	Age-adjusted mortality rate^b^	YLL^b,c^	Crude YLL rate^b,c^	Age-adjusted YLL rate^b,c^ (95%CI)
		
			(95%CI)	(95%CI)		(95%CI)	
2009	193,543,969	7,060	3.65 (3.56–3.73)	4.01 (3.91–4.10)	207,712.70	107.32 (106.86–107.78)	111.44 (110.96–111.92)
2010	194,890,682	7,951	4.08 (3.99–4.17)	4.25 (4.15–4.34)	231,847.40	118.96 (118.48–119.45)	121.46 (120.97–121.96)
2011	196,603,732	8,349	4.25 (4.16–4.34)	4.33 (4.24–4.42)	251,214.20	127.78 (127.28–128.28)	128.94 (128.43–129.45)
2012	198,314,934	8,947	4.51 (4.42–4.60)	4.55 (4.46–4.65)	270,542.20	136.42 (135.91–136.94)	137.53 (137.01–138.05)
2013	200,004,188	9,535	4.77 (4.67–4.86)	4.73 (4.64–4.83)	292,143.10	146.07 (145.54–146.60)	145.98 (145.45–146.52)
2014	201,717,541	9,906	4.91 (4.81–5.01)	4.79 (4.70–4.89)	301,254.30	149.34 (148.81–149.88)	148.08 (147.55–148.62)
2015	203,475,683	10,376	5.10 (5.00–5.20)	4.90 (4.80–4.99)	309,070.50	151.90 (151.36–152.43)	149.19 (148.66–149.72)
2016	205,156,587	10,630	5.18 (5.08–5.28)	4.90 (4.80–4.99)	314,827.50	153.46 (152.92–153.99)	150.12 (149.59–150.64)
2017	206,804,741	10,996	5.32 (5.22–5.42)	4.95 (4.86–5.04)	324,887.70	157.10 (156.56–157.64)	152.46 (151.94–152.99)
2018	208,494,900	11,481	5.51 (5.41–5.61)	5.06 (4.97–5.15)	339,913.00	163.03 (162.48–163.58)	157.92 (157.39–158.46)
Average	-	9,523.1	4.73 (SD = 0.60)	4.65 (SD = 0.35)	284,341.3	141.14 (SD = 18.04)	140.31 (SD = 15.10)
		(SD = 1,427.94)	(95%CI: 4.30–5.15)^d^	(95%CI: 4.40–4.90)^d^	(SD = 42,863.42)	(95%CI: 128.23–154.04)^d^	(95%CI: 129.51–151.11)^d^
APC	-	7.4 (5.5–9.2)*	6.5 (4.6–8.4)*	4.1 (3.0–5.2)*	8.5 (7.1–10.0)*	7.7 (6.3–9.1)*	6.7 (5.5–7.9)*
(2009–2013)							
APC	-	3.6 (2.5–4.7)*	2.7 (1.4–4.0)*	1.3 (0.5–2.0)*	2.8 (2.0–3.6)*	1.9 (1.0–2.8)*	1.3 (0.5–2.1)*
(2004–2018)							
AAPC	-	5.2 (4.5–6.0)*	4.4 (3.5–5.2)*	2.5 (2.0–3.0)*	5.3 (4.7–5.9)*	4.4 (3.8–5.0)*	3.7 (3.1–4.2)*
(2009–2018)							

ADE: adverse drug event; APC: annual percent change; AAPC: annual average percent change; 95%CI: 95% confidence interval; SD: standard deviation; YLL: years of life lost.

* Indicates that the APC and AAPC are significantly different from zero at the alpha level (0.05).

^a^ According to *IBGE* (2013 and 2018 projections).

^b^ 101 registries with missing data on age were excluded.

^c^ For 14 registries with missing data on gender, the life expectancy for both genders was considered.

^d^ t.test.

**Figure 1:**
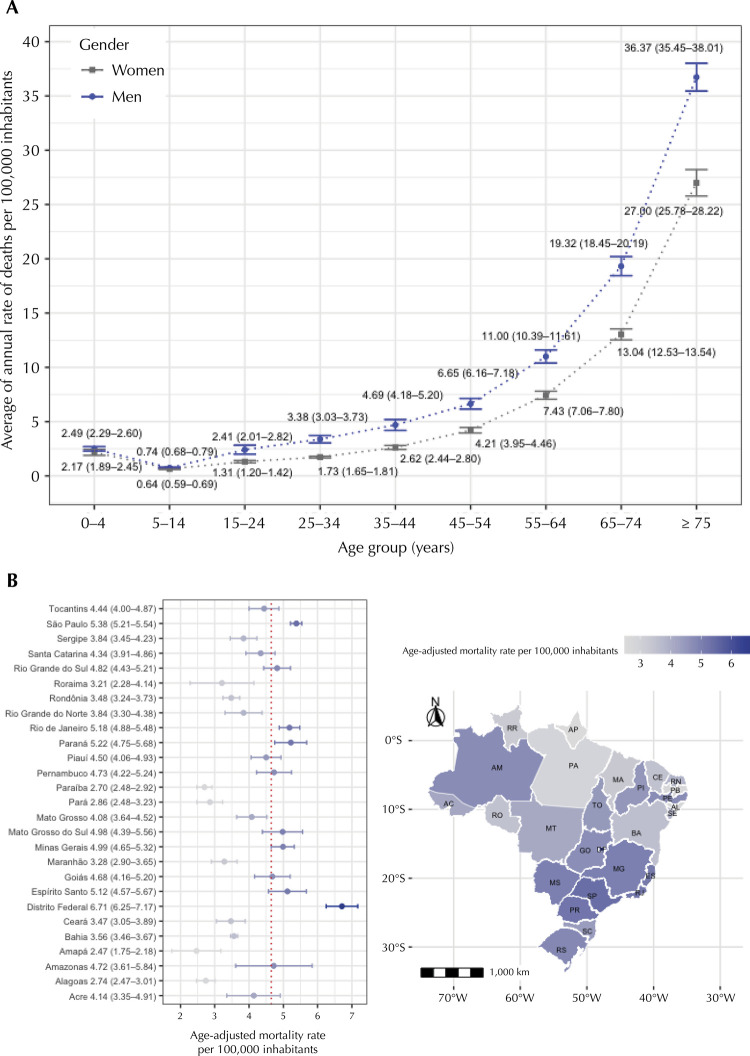
(A) Gender- and age-specific mortality rates related to adverse drug events per 100,000 inhabitants and (B) Mortality rates related to adverse drug events per 100,000 inhabitants by Brazilian Federative Unit. Brazil, 2009–2018.

Figure 1A shows the average annual gender- and age-specific mortality rates per 100,000 inhabitants. The highest gender- and age-specific overall mortality rate was observed in men aged ≥ 75 years (36.73 per 100,000 inhabitants, 95%CI: 35.45–38.01). Gender- and age-specific mortality rates increased in all age groups over the years, with the highest annual growth observed in men aged 15–24 years (7.8%). Figure 1B shows the geographical variation in mortality rates related to ADEs by Brazilian Federative Unit. The average mortality rate was higher in 11 units compared to the national average rate. There was a 2.8-fold difference between the unit with the highest (Distrito Federal) and lowest (Amapá) average annual mortality rates (see supporting information)^a^.

### Years of Life Lost

A total of 2,843,413 YLL were attributed to deaths related to ADEs throughout the period (1,600,077 YLL for men and 1,242,590 for women—gender identification was not possible in 14 cases). When considering only the underlying cause of death, 984,375 YLL were identified. The mean number of YLL per year was 284,341.3 (SD = 42,863.42), and the average number of YLL per death was 29.89 (SD = 18.42) over the ten-year period (Table 2). Deaths in the 35-44 years age group contributed most to the overall number of YLL (YLL = 425,619.8; 14.97%).

**Figure 2:**
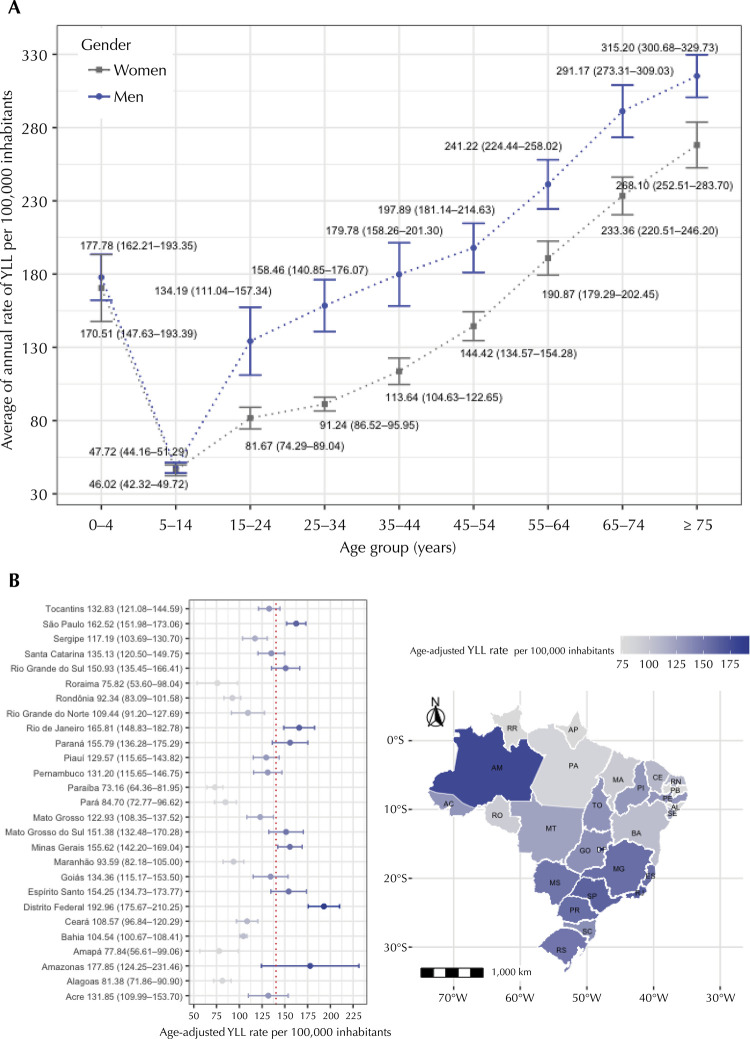
(A) Gender- and age-specific rates of years of life lost related to adverse drug events per 100,000 inhabitants and (B) Rates of years of life lost related to adverse drug events per 100,000 inhabitants by Brazilian Federative Unit. Brazil, 2009–2018.

The average annual crude YLL rate per 100,000 inhabitants was 141.14 (95%CI: 128.23–154.04). The highest age-adjusted YLL rate was observed in 2018 (157.92 per 100,000 inhabitants, 95%CI: 157.39–158.46) (Table 2), with an annual increase of 3.7% in the rate over the years. Figure 2A shows the specific YLL rates per 100,000 inhabitants, considering gender and age groups. Deaths in older age groups produced higher YLL rates in both genders. For all age groups and both genders, YLL rates increased over the years. Figure 2B shows the geographical distribution of YLL per 100,000 inhabitants by Brazilian Federative Unit. The average YLL rate was higher in nine Federative Units compared to the national average rate (see supporting information)^a^.

## DISCUSSION

We conducted a population-based analysis in Brazil over a ten-year period, which revealed a relatively high number of deaths (95,231) and YLL (2,843,413) associated with ADEs, which translated into a substantial average age-adjusted mortality rate (4.65) and YLL rate (140.31) per 100,000 inhabitants. In our analysis, we found that important variables such as gender and age were missing from the register in only a few cases, which allowed us to obtain more comprehensive information. We were systematic in capturing all deaths with at least one ADE-related ICD-10 code in a large database and assessed national mortality and YLL rates to provide evidence of the significant health burden associated with ADEs. Our analysis contributes to the literature on drug safety, which has been growing since the Institute of Medicine report in 1999 and is in line with the world health agenda^20^.

For the overall proportion of ADEs, our results were higher when compared to those of other studies. In high-income countries such as the US, one study found a proportion of 0.20% of deaths related to ADRs, a specific type of ADEs^21^. In Sweden, when authors expanded the results of a study that only included fatal ADRs to the entire country, these events were found to be the seventh most common cause of death^22^. In Brazil, 0.1% of deaths from 2000 to 2014 were related to drug poisoning (regardless of intention and excluding codes of external cause with questionable interpretation regarding legal and illegal drugs) and ADRs^23^. Mota et al.^13^, considering all types of drug poisoning from 1996 to 2005, found a proportion of 0.04% of deaths associated with this outcome in Brazil. The percentage of ADE-related deaths (0.78%) found in our study represents a higher threshold. The differences found between these studies can be partially explained by the definition of ADE used, the method of identification (e.g. ICD codes selected), and the characteristics of the population. These differences make direct comparations between studies on the prevalence of deaths related to ADEs difficult. Our analysis used a wide range of ICD-10 codes, including those such as “due to drugs,” “drug-induced,” and “secondary to drugs,” and not only the external cause of death. In addition, we analyzed deaths with an ICD-10 codes of interest, regardless of their presence in the underlying or contributing cause of death.

In our study, we observed an annual increase of 2.5% in overall drug-related mortality rates from 2009 to 2018, with a greater increase in rates among males aged 15 to 24 years. Regarding YLL rates, the increase was approximately 3.7%. An annual upward trend in the prevalence and rates of ADE-related deaths has been demonstrated in many studies^4,5,23^. Santos and Boing^23^ showed an almost twofold increase in death rates due to ADE in Brazil over a 15-year period. An increase in the number of deaths due to ADEs is expected over time, considering various aspects, such as the introduction of new drugs to the market each year and the prescription/use of many drugs by patients, which results in different categories of drug interactions (major, moderate, and minor). In addition, improvements in databases records and the coding system may have contributed to the trends^24^.

Men contributed more significantly to the number of deaths and YLL than women in general (~ 56% for both outcomes) and in all age groups, which is consistent with previous studies^5,21,25^. The effect of ADEs was observed in all age groups. However, higher rates were found in older age groups. Wester and colleagues studied only fatal ADRs and showed that most of these events occurred in individuals aged over 60 years^25^. Older age is associated with a higher likelihood of ADEs, presumably due to polypharmacy, greater disease complexity, and age-related pharmacokinetic and pharmacodynamic changes^25^.

There was a 2.8-fold difference between the Brazilian Federative Units with the highest and lowest average annual rates of deaths related to ADEs and a 2.6-fold difference regarding YLL rates. These relative differences in rates suggest that there are factors influencing the magnitude of this variation. One possible explanation for this variation is the difference in regional prescription/use/access patterns, which can lead to adverse consequences such as increased mortality^28^. In addition, factors such as differences in the interpretation and application of ICD-10 coding rules at the local level can influence the coding process. Information on the regional variation in the annual rate of ADE-related mortality is scarce in the literature, and the interstate variability highlighted in this report underscores the need to better understand the factors that influence regional differences^29^.

In addition to mortality, we used YLL as an outcome to consider the age at death, as mortality only accounts for the number of deaths. This approach allows for a more rational assessment of the impact of deaths related to ADEs since YLL take into account life expectancy. We calculated an average of 29.89 YLL per person during the 10-year period studied, which is an interesting parameter as it provides a measure of the burden of events on individuals rather than the population. Our results indicated that deaths among individuals aged 35 to 44 years old contributed the most to the total YLL. These findings emphasize that each death results in a significant number of years of life lost, which underscores the impact of ADEs on individuals, including a significant proportion of the economically active population^12,30^.

### Strengths and Limitations

We included data from a comprehensive database with complete national coverage, which is used in public health management to identify health problems and establish priorities. We believe that the length of the study period and the extensive data provided by the SIM offer sufficient internal validity, given the consistent frequency of episodes detected each year, by age groups and genders. This allowed us to compile reliable population-based data for our analysis and generate a robust metric in epidemiology.

However, the data accuracy is limited by the constraints of the health system, which depends on the precision of data collection. The level of accuracy in coding cases of death is susceptible to errors, variation between codifiers, and human mistakes. In addition, under-recording of ADEs can occur due to the complexity of recognizing them. Another limitation of this study is related to the difficulties of making comparisons with other studies due to differences in outcomes and the use of ICD codes. The limitations associated with terminology hinders the analysis of the data reported in databases, as ADEs include a wide range of harm types and concepts. One factor that contributed to this limitation is which fields of death certificates were considered in the analysis. We used the underlying cause of death and all contributing causes, rather than only the underlying cause. Limitations related to the structure of ICD-10 itself must be imputed. Some codes encompass both legal and illegal drugs, making it challenging to impose such restrictions. In addition, some codes that are not always related to drugs were included, because drugs have been identified as a significant etiological factor in these diseases.

In conclusion, this nationwide epidemiological study provides an overview of population and geographic rates of ADE occurrence in terms of deaths and YLL, using a Brazilian vital statistic system. We observed increasing trends in deaths and YLL over the ten-year period studied. The findings of this study emphasize the importance of using information on multiple causes of death from death certificates to monitor the occurrence of country- and region-specific deaths and YLL related to ADEs in the public health practice. This information is crucial for planning national-level strategies to understand the variations found between genders, age groups, and Federative Units, which can inform policy recommendations at a higher level.

## Footnotes

a Available at: https://redcap.prpi.ufg.br/surveys/?__file=MKhb7AKLkPeqv6jIhxwfhCxCTjLxMM3inkcvgVdCCDvU2ZT46mXHX7UgWciJa3d8K9QpZn29Xxkv7knCeDijqDvUGyXj7uKYtdj5
